# Unexpected Increase of Alveolar Echincoccosis, Austria, 2011

**DOI:** 10.3201/eid1903.120595

**Published:** 2013-03

**Authors:** Renate Schneider, Horst Aspöck, Herbert Auer

**Affiliations:** Author affiliation: Medical University Vienna, Vienna, Austria

**Keywords:** Alveolar echinococcosis, incidence, Austria, helminth, parasites, Echinococcus multilocularis, tapeworm

## Abstract

Austria is part of the classical area of central Europe to which alveolar echinococcosis (AE) is endemic. Annual incidences in Austria were 2.4 and 2.8 cases/100,000 population during 1991–2000 and 2001–2010, respectively. Hence, the registration of 13 new AE patients in 2011 was unexpected. Increasing fox populations and past AE underreporting might have caused this increase.

Alveolar echinococcosis (AE) is one of the most serious helminthic diseases of humans. It is caused by the larval stages (metacestodes) of the fox tapeworm, *Echinococcus multilocularis*. Final hosts are foxes and, rarely, dogs and cats; intermediate hosts are rodents (voles). Humans are aberrant hosts and acquire the infection by oral ingestion of parasite eggs released in the feces of infected foxes or other carnivores. The metacestodes proliferate in the human liver and induce a hepatic disorder mimicking liver cancer (*1*) that becomes clinically apparent after an incubation period of 5–15 years. The prognosis for untreated AE is poor, and early diagnosis is essential for curative treatment (*2*).

AE has been known to be endemic to Austria, southern Germany, Switzerland, and eastern France since the second half of the nineteenth century. From the Austrian echinococcosis researcher, Adolf Posselt, who documented all reported human AE cases during 1867–1936, we know that the annual incidence of AE in Austria was 1.4 cases at the beginning of the twentieth century and that most patients resided in Austria´s western provinces, Vorarlberg and Tyrol (*3*).

Meanwhile, the parasite and the disease have spread from its classical distribution area in central Europe to at least 11 other European countries (*2**,**4*). Furthermore, several reports from Austria, Germany, and Switzerland document increasing fox populations during the 1990s and 2000s, presumably caused by successful antirabies vaccination (*2**,**5*), which was established in Austria in 1992. In addition, a suspected increase in and/or underreporting of human AE has been discussed within the past few years (*6*,*7*).

The goal of our study was to determine the annual incidence of AE during the past 20 years. We also aimed to discuss possible reasons for the unexpected increase of human AE during 2011.

## The Study

Our institute is Austria`s national reference center for echinococcosis (*8*). The study comprised 65 patients in whom AE was diagnosed and registered during 1991–2011; the patients derived from all 9 of Austria’s provinces. Inclusion criteria were as follows: 1) AE characteristic imaging findings and 2) *E. multilocularis*–positive species-specific serology and/or 3) AE characteristic histopathologic findings and species-specific molecular analysis. All available details about sex, age, province of origin, results of serologic and molecular biologic investigations, histopathologic findings, and clinical status of AE patients before AE diagnosis were logged into an Excel spreadsheet (Microsoft, Redmond, WA, USA) and analyzed.

Until 2000, we used an in-house crude-antigen ELISA for serologic screening and an in-house immunoblot for species-specific diagnosis (*9*,*10*). In 2000, the in-house immunoblot was replaced by a commercially available immunoblot (LDBIO) (*11*). In 2004, a species-specific PCR was established that detects *E. multilocularis*–specific DNA from fresh or formalin-fixed paraffin-embedded tissue; this method was also applied to retrospectively confirm suspected cases of AE (*12*). To outline the development of the annual incidence of AE in Austria in general and in Vorarlberg and Tyrol Provinces in particular, we compared the annual incidence (patients/year) and the annual incidence rates (patients/100,000 population/year) for 1991–2000 (24 patients) and 2001–2010 (28 patients) with those for 2011 (13 patients) (Table).

**Table:** Incidences of alveolar echinococcosis, Austria, 1991–2011

Variable	Observation period		
	1991–2000	2001−2010	2011
Location, no. patients (cases/100,000 population/year)			
Austria, n = 8,217,280 residents*	24 (0.029)	28 (0.034)	13 (0.158)
Tyrol Province, n = 710,048 residents*	12 (0.17)	5 (0.07)	4 (0.56)
Vorarlberg Province, n = 372,000 residents*	3 (0.08)	12 (0.32)	7 (1.9)
Patient characteristics			
Average age (range), y	50.5 (7–78)	56.5 (37–80)	68.6 (45–90)
Sex, no. (%)			
F	12 (50)	10 (36)	6 (46)
M	12 (50)	18 (64)	7 (54)
Asymptomatic, no. (%)	10 (42)	11 (39)	5 (38)

*Census 2011.

During 1991–2000, a total of 24 human AE cases were diagnosed (mean incidence 2.4 new cases/year). The patients derived mainly from the western provinces, Vorarlberg (3 [13%] patients) and Tyrol (12 [50%] patients).

During 2001–2010, we diagnosed 28 AE cases (mean incidence 2.8 new cases/year). The patients derived mainly from Vorarlberg (12 [43%] patients) and Tyrol (5 [18%]).

In contrast to former decades, we registered 13 new AE cases during 2011 (Figure; Table). Four patients resided in Tyrol, and 7 resided in Vorarlberg.

**Figure:**
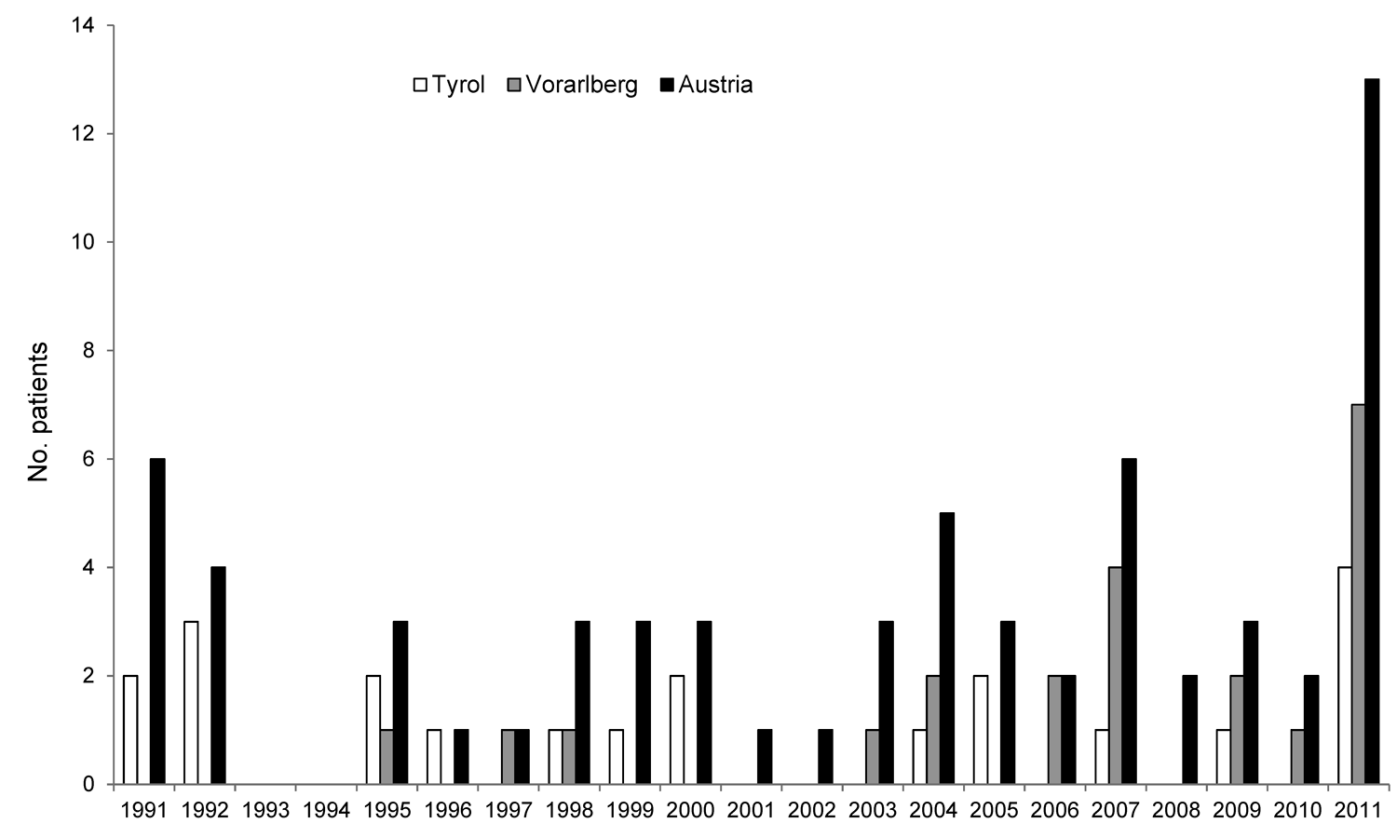
Incidence of alveolar echinococcosis, Austria and its provinces of Tyrol and Voralberg, 1991–2011.

The epidemiologic situation for AE changed most significantly in the most western province (Vorarlberg) during the observation period. The annual incidence rate in Vorarlberg was 0.08 cases per 100,000 population during 1991–2000; it increased to 0.32 cases per 100,000 population during 2001–2010 and peaked in 2011 at 1.9 cases per 100,000 population (Table). Vorarlberg, with 372,001 residents, is one of least populated Austrian provinces. In Tyrol (710,048 residents) the annual incidence rate varied from 0.17 (1991–2000) to 0.07 (2001–2010) to 0.56 per 100,000 population in 2011 (Table). The other 7 provinces showed only sporadic cases during the study period.

## Conclusions

Because of the long incubation period of human AE (5–15 years) constant observation of the annual incidence and geographic distribution is critical (*13*). The annual incidence of 2.4 cases (1991–2000) and 2.8 cases (2001–2010) in the past 2 decades represents a moderate increase from the 1.4 cases at the beginning of the twentieth century. Hence, the 13 new cases in 2011 were unexpected, and we assume the following reasons for this increase.

We know from studies in Switzerland and Poland that increasing fox populations changed their population dynamics and live in proximity of villages or even cities (*6*,*14*). The general increase in AE incidence in Austria is mainly due to the increase in Vorarlberg. Duscher et al. (*5*) reported Vorarlberg as the Austrian province with the highest prevalence of *E. multilocularis*–infected red foxes (up to 60%); this high prevalence is a prerequisite for human AE. Although the high incidence in 2011 (7 new cases in Vorarlberg) of such a rare disease can be misinterpreted, data from the past 21 years show a continuous increase of cases in Vorarlberg (Table; Figure). Such an increase in some regions also was observed in neighboring Switzerland (e.g., the Swiss Jura [0.75 cases/100,000 population]) (*13*).

Past underreporting of AE is a problem in Austria (and other European countries) and could partially explain the suddenly increased number of cases (*7*). Although human AE has been reportable in Austria since 2004, only 2 of the 13 cases from 2011 were reported to the Ministry of Health. Our recent data indicate that AE diagnosed only histopathologically is not reported by pathology institutes, a presumption suggested by Jorgerson et al. (*7*),

In addition, our institute provides newly established molecular biologic methods (PCR) enabling not only an exact differentiation between liver cancer or metastases and AE but also species-specific diagnosis based on native material from operations on AE patients and on formalin-fixed paraffin-embedded tissue (*12*). These methods proved to be valuable diagnostic tools.

Because the number of asymptomatic patients remained almost stable during the study period (Table), there is no evidence to suggest that the stage at which AE is diagnosed today is earlier than in past decades. We share this observation with neighboring Switzerland (*6*).

In conclusion, we assume there are several reasons (i.e., increasing fox population, past underreporting, more sensitive laboratory diagnostic tools) for the increasing number of AE cases in Austria within the past decades and especially in 2011. The next years will show whether the high incidence in 2011, which is in accordance with a study from neighboring Switzerland (*6*), is a statistical outlier or reflects a persistent event. We propose that the surveillance system in Austria be improved. Hence, we suggest that serologic screening of exposed groups, such as hunters or farmers, in *E. multilocularis*–endemic regions, such as Vorarlberg, with increasing fox populations could lead to some early diagnosed and therefore successfully treated AE cases (*15*).
